# Does Complementary Shoulder and Hand Ultrasound in the Diagnosis of Patients with Polymyalgia Rheumatica Differ in Clinical Course After Glucocorticoid Induction Compared to After a Simple Clinical Diagnosis?

**DOI:** 10.14789/jmj.JMJ24-0020-OA

**Published:** 2024-09-11

**Authors:** TOMOHIRO KAWAGUCHI, MICHIHIRO OGASAWARA, TOSHIO KAWAMOTO, YUKO MATSUKI-MURAMOTO, KEN YAMAJI, NAOTO TAMURA

**Affiliations:** 1 Department of Internal Medicine and Rheumatology, Juntendo University Faculty of Medicine, Tokyo, Japan; 1 Department of Internal Medicine and Rheumatology, Juntendo University Faculty of Medicine, Tokyo, Japan

**Keywords:** polymyalgia rheumatica, ultrasound, clinical course, glucocorticoid

## Abstract

**Background/Objective:**

Patients with polymyalgia rheumatica experience flares and require a lengthy course of glucocorticoid treatment. Ultrasound application is often used for diagnosing polymyalgia rheumatica. This study aimed to determine whether polymyalgia rheumatica diagnosed with ultrasound complementation has a more favorable clinical course compared with that of only clinically diagnosed patients.

**Methods:**

In this cohort study, we retrospectively identified 152 patients with polymyalgia rheumatica from January 2008 to December 2018. We extracted patients' clinical and ultrasound information, and hazard ratio and propensity-score matched analyses were performed.

**Results:**

Among 152 patients with polymyalgia rheumatica, the flare, methotrexate add-on, and C-reactive protein normalization rates were 15.9 (95% confidence interval, 8.8-23.1)/100 person-years, 9.3 (3.6-15.0) /100 person-years, and 70.3 (61.3-79.2) /100 person-months, respectively. Age (p=0.01), C-reactive protein levels (p=0.03), and absence of peripheral joint pain (p=0.03) were significantly different between 81 and 71 patients with and without ultrasound complementation, respectively. The hazard ratio showed that ultrasound complementation did not contribute to the clinical course; flare, methotrexate add-on, and C-reactive protein level normalization yielded values of 0.88 (p=0.64), 1.93 (p=0.056), and 0.94 (p=0.72), respectively. Propensity-score-matched analysis showed a similar clinical course between 51 pairs: flare (p=0.45), methotrexate add-on (p=0.15), and C-reactive protein normalization (p=0.94).

**Conclusions:**

Age, C-reactive protein, and involved joint distribution were factors leading to ultrasound complementation at the time of polymyalgia rheumatica diagnosis. Ultrasound complementation at PMR diagnosis is useful for differential diagnosis but may not affect the clinical course after GC introduction.

## Introduction

Differentiating polymyalgia rheumatica (PMR) from elderly onset rheumatoid arthritis (RA) is often difficult based on clinical examination and blood test results alone, and complementary imaging studies such as ultrasound are effective in improving the accuracy of differential diagnoses^1-3)^. On the other hand, there are limited reports on the predictive ability and usefulness of complementary ultrasound at the time of PMR diagnosis for the clinical course of PMR after initiation of glucocorticoid (GC) therapy^4-6)^.

Prolonged GC therapy is an issue that needs to be addressed because most patients with PMR experience recurrence and are at increased risk for GC-related side effects^7)^. Although female sex, high erythrocyte sedimentation rate (ESR), and peripheral joint involvement have been reported as poor prognostic factors for the course of treatment in PMR^8)^, the utility of baseline ultrasound complements for the course of treatment should also be investigated.

Ultrasound increases the specificity of PMR diagnosis, helps differentiate PMR from mimicking forms of the disease^1, 9, 10)^, and plays an important role in selecting patients with PMR with greater accuracy than a simple clinical diagnosis. Some reports have examined the association between ultrasound findings and clinical course, including recurrence and GC reactivity^4, 11)^, and others have found no association between ultrasound findings and recurrence or GC reactivity^6, 12)^. However, there are no studies on whether the presence or absence of complementation by ultrasound findings in PMR diagnosis affects the clinical course after GC treatment.

Therefore, this study examined the effect of PMR diagnosis with ultrasound assistance on the clinical course of PMR patients after GC treatment. A comparative analysis of the clinical course of patients diagnosed with PMR without ultrasound assistance was retrospectively performed.

## Methods

### Patients

**Figure 1:**
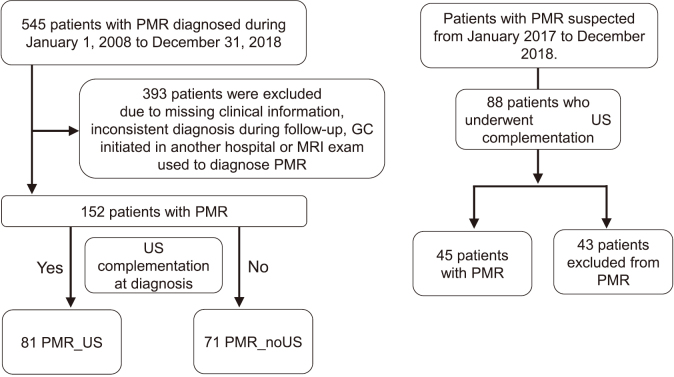
Flow chart showing the patient selection process PMR, polymyalgia rheumatica; MRI, magnetic resonance imaging; US, ultrasound; PMR_US, PMR patients diagnosed with ultrasound complementation; PMR_noUS, PMR patients diagnosed without ultrasound complementation

We conducted a single-center, retrospective, observational cohort study. We enrolled patients diagnosed with PMR in our department from January 2008 to December 2018. The diagnosis was based on each attending rheumatologist's judgment. We collected patients' blood test results and clinical information including items in the criteria (Bird^13)^ and EULAR/ACR^5)^). Blood tests included C-reactive protein (CRP), ESR, anti-cyclic citrullinated peptide, and rheumatoid factor. Only PMR patients except for giant cell arteritis with a consistent diagnosis during follow-up and who started GCs in our department were selected in the following analysis. We also excluded PMR patients examined and diagnosed with magnetic resonance imaging (MRI). We classified selected patients into two groups: (1) PMR patients diagnosed with ultrasound complementation (PMR_US), or (2) PMR patients diagnosed without ultrasound complementation (PMR_noUS) (Figure 1).

In addition, PMR-suspected patients who underwent ultrasound from January 2017 to December 2018 were selected to identify other kinds of diseases that should be excluded from the diagnosis of PMR (Figure 1).

### Endpoints

We compared the clinical course, including flare, concomitant usage of methotrexate (MTX add-on) as a steroid-sparing agent, and inflammatory marker improvement (CRP normalization) rates between the PMR_US and PMR_noUS groups. The endpoints were set to the time from GC administration to flare, MTX add-on, or CRP-negative status. A flare was defined as an increase in GC dose and/or the documentation of flare. The flare-free duration was calculated from the day of GC administration to the day of flare. A CRP level under 0.3 mg/dL was defined as negative, CRP normalization. Days to MTX introduction were calculated from the day of GC administration to the day of MTX introduction. All patients' treatment course depended on their attending physician.

### Ultrasound examination

**Figure 2:**
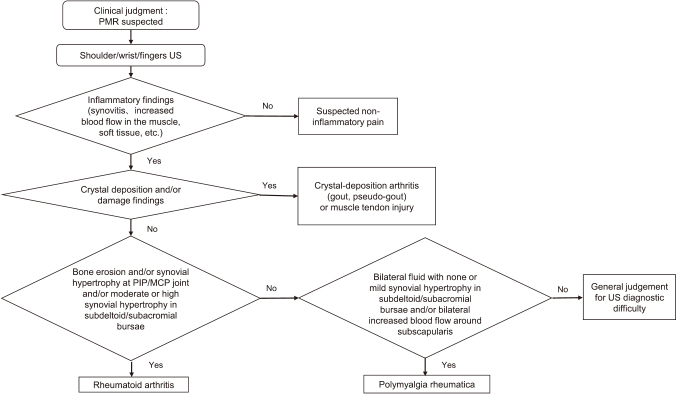
Ultrasound examination flow PMR, polymyalgia rheumatica; US, ultrasound; PIP, interphalangeal; MCP, metacarpophalangeal

Patients in the PMR_US group underwent ultrasound examination on both hands and shoulders. The examinations were performed using an ARIETTA 70 ultrasound unit (FUJIFILM Healthcare Co., Ltd.) equipped with a high-frequency linear transducer L64 (5-18 MHz) by three rheumatologist sonographers with at least 5 years of experience (>2500 examinations) in musculoskeletal ultrasound (MO, TK, YM) who were blinded to the clinical examination and diagnosis as well as to the radiographic imaging findings. Scanning was conducted according to the EULAR standardized procedures^14)^. Longitudinal and transverse scans were performed using both B-mode and Doppler mode. The Doppler gain was set at the level just below random noise; the pulse repetition frequency was adjusted between 0.5 and 0.8 MHz throughout the study. All joints were examined in the neutral relaxed position. Examinations were performed in a temperature-controlled dark room. The ultrasound experts excluded other diseases, such as non-inflammatory pain, crystal-deposition arthritis, muscle tendon injury, and rheumatoid arthritis (RA), before issuing the examination reports following the general ultrasound examination flow (Figure 2).

### Statistical analyses

Data were censored on January 1, 2020. Data for patients who were lost to follow-up were censored on the day of their last visit. The number of cases in this study duration decided the sample size. Our missing data analysis methods operated missing at random assumptions. We used the chained equations method for multiple multivariate imputation. In the multivariate logistic regression analyses, we analyzed 10 copies of the data independently, and every missing value was imputed appropriately. To determine differences between the groups, the t-test was used for independent groups, and the chi-square test was used for proportions of baseline data.

For time-to-event endpoints, days to CRP-negative status, days to MTX introduction, and days to flare, the adjusted hazard ratio was calculated using the Cox proportional hazard model to analyze the influence of ultrasound complementation in the diagnosis of PMR, adjusting for sex, age, baseline CRP levels, and involved joint distribution, which were reported as potential prognostic factors^7)^.

A propensity-score-based matching method was used to select PMR_US and PMR_noUS to be directly compared. The propensity score, a predicted probability of each PMR patient to be complemented with ultrasound, was calculated using logistic regression on ultrasound examination (outcome) with covariates including all possible confounders (sex, age, items in Bird and EULAR/ACR criteria, and CRP). Each PMR patient who received ultrasound examination was matched to a PMR patient who did not receive ultrasound examination with the nearest propensity score. Kaplan-Meier survival curves were estimated separately for PMR_US and PMR_noUS groups and compared using the log- rank test.

All statistical analyses were conducted using R, version 4.1.2 (R Foundation for Statistical Computing) and EZR^15)^. The threshold of significance was set at p<0.05. This study was conducted in accordance with the Declaration of Helsinki and was approved by the ethics committee of Juntendo University (No.19-153); the study plan and opt-out forms were available on the hospital website. The requirement for consent was waived because this was an IRB-approved retrospective study.

## Results

### Patient characteristics

The study population was selected from all patients with PMR in this cohort (n=545) who fulfilled the inclusion criteria during the study period. A total of 152 patients with PMR were identified. The patient baseline characteristics are shown in Table 1.

For all the studied patients, the flare rate was 15.9 (95% confidence interval [CI], 8.8-23.1) /100 person-years, the rate of MTX add-on was 9.3 (95% CI, 3.6-15.0) /100 person-years, and the rate of CRP normalization was 70.3 (95% CI, 61.3-79.2) /100 person-months. The event-free survival rate and 50% survival duration estimates are shown in Table 2.

Of the 152 PMR patients, 81 were in the PMR _US group, and the remaining 71 were in the PMR_noUS group. Age (p=0.01), CRP level before treatment (p=0.03), and absence of other joint pain (p=0.03) were significantly different between the groups. The percentage of patients who met the ACR/EULAR and/or Bird criteria and the initial dose of GC were comparable between the groups (Table 1).

**Table 1:** Baseline characteristics of PMR patients

	Total PMR (n=152)	PMR_US (n=81)	PMR_noUS (n=71)	p-value	Propensity-score matched		
					PMR_US (n=51)	PMR_noUS (n=51)	p-value
Age, years, mean (SD)	70.6 ± 9.2	72.3 ± 8.3	68.7 ± 9.9	0.01	70.5 ± 9.6	70.9 ± 8.1	0.82
Female sex, n (%)	101(66.4)	56(69.1)	45(63.4)	0.49	34(66.7)	36(70.6)	0.83
Bird criteria							
Shoulder pain and/or stiffness bilaterally, n (%)	149(98.0)	81(100.0)	68(95.8)	0.09	51(100.0)	51(100.0)	NA
Onset of illness of <2 weeks duration, n (%)	67(44.0)	40(49.4)	27(38.0)	0.19	22(43.1)	22(43.1)	1
Initial ESR ≥40 mm, n (%)	145(95.3)	77(95.1)	68(95.8)	1	49(96.1)	49(96.1)	1
Morning stiffness duration >1 h, n (%)	68(44.7)	36(44.4)	32(45.1)	0.49	26(51.0)	25(49.0)	1
Age >65 years, n (%)	114(75.0)	65(80.2)	49(69.0)	0.13	39(76.5)	40(78.4)	1
Depression and/or loss of weight, n (%)	39(25.6)	23(28.4)	16(22.5)	0.46	13(25.5)	12(23.5)	1
Upper arm tenderness bilaterally, n (%)	114(75.0)	60(74.1)	54(76.1)	0.85	39(76.5)	40(78.4)	1
Total score	4.57 ± 1.19	4.72 ± 1.14	4.42 ± 1.24	0.13			
Criteria fulfillment, n (%)	148(97.3)	80(98.8)	68(95.8)	0.34			
							
ACR/EULAR criteria							
Morning stiffness duration >45 min, n (%)	73(48.0)	38(46.9)	35(49.3)	0.32	21(41.2)	24(47.1)	0.45
Hip pain or limited range of motion, n (%)	109(71.7)	56(69.1)	53(74.6)	0.47	41(80.4)	37(72.5)	0.48
Absence of other joint pain, n (%)	28(18.4)	20(24.7)	8(11.3)	0.03	7(13.7)	8(15.7)	1
Absence of RF or ACPA, n (%)	132(86.8)	72(88.9)	60(84.5)	0.47	45(88.2)	48(94.1)	0.48
Total score	3.66 ± 1.33	3.78 ± 1.42	3.54 ± 1.22	0.26			
Criteria fulfillment, n (%)	79(51.9)	44(54.3)	35(49.3)	0.62			
							
Level of CRP before treatment (mg/dL)	6.7 ± 4.7	7.8 ± 5.2	5.9 ± 4.0	0.03	6.0 ± 4.3	5.8 ± 3.2	0.79
Initial dose of GC (mg)	13.7 ± 4.4	13.6 ± 4.4	13.8 ± 4.3	0.66	13.6 ± 4.1	13.9 ± 4.3	0.72

Data are shown as count (%) or mean ± SD. Comparisons were performed using the chi-square test and t-test. PMR, polymyalgia rheumatica; US, ultrasound; ESR, erythrocyte sedimentation rate; RF, rheumatoid factor; ACPA, anti-cyclic citrullinated peptide antibody; CRP, C-reactive protein; GC, glucocorticoid; SD, standard deviation

**Table 2:** Clinical course of PMR

	Total cases (n=152), n (%)	Person-years	Incidence rate/100 person-years (95% CI)	One-year event-free survival rate (95% CI)	50% survival years, median (95% CI)
Flare	61 (40.1)	381.3	15.9 (8.8-23.1)	82.7 (75.1-88.2)	4.4 (3.0-6.6)
MTX add-on	40 (26.3)	428.0	9.3 (3.6-15.0)	78.1 (70.2-84.0)	NA (NA-NA)
		Person-months	Incidence rate/100 person-months (95% CI)	One-month event-free survival rate (96% CI)	50% survival weeks, median (96% CI)
CRP normalization	144 (94.7)	204.7	70.3 (61.3-79.2)	37.5 (29.7-45.3)	3.7 (3.0-4.0)

PMR, polymyalgia rheumatica; CI, confidence interval; NA, not available; MTX, methotrexate; CRP, C-reaction protein

### Differential diagnosis from PMR with ultrasound complementation

In total, 88 patients with clinically suspected PMR underwent ultrasound evaluation from January 2017 to December 2018, for a differential diagnosis (Figure 1). Among them, 43 (48.8%) patients were excluded from the diagnosis of PMR as follows: 22 for RA, 1 for gout, 1 with pseudo-gout, 1 with remitting seronegative symmetrical synovitis with pitting edema, 1 with psoriatic arthritis, 1 with Sjogren's syndrome, and 16 with non-inflammatory pain.

### Hazard ratio analysis

Hazard ratio estimates suggested that ultrasound complementation did not contribute to the clinical course. Hazard ratios were 0.88 (95% CI, 0.51-1.50, p=0.64) in flare, 1.93 (95% CI, 0.98-3.81, p=0.056) in MTX add-on, and 0.94 (95% CI, 0.66-1.32, p=0.72) in CRP normalization. Age, female sex, absence of other joint pain, and ESR did not contribute to these primary endpoints (Table 3).

**Table 3:** Impact of ultrasound complementation contributing to the clinical course

Outcome measures	Hazard ratio		p-value
Relapse	(95% CI)		Value
Ultrasound complementation	0.88	(0.51-1.5)	0.64
Age	0.99	(0.96-1.02)	0.54
Female sex	0.98	(0.55-1.74)	0.95
Absence of other joint pain	1.23	(0.65-2.31)	0.51
Initial ESR ≥40 mm	1.45	(0.33-6.24)	0.61
MTX add-on	(95% CI)		p-value
Ultrasound complementation	1.93	(0.98-3.81)	0.056
Age	0.96	(0.93-1.00)	0.060
Female sex	1.17	(0.58-2.36)	0.65
Absence of other joint pain	0.99	(0.44-2.20)	0.98
Initial ESR ≥40 mm	0.64	(0.19-2.20)	0.48
CRP normalization	(95% CI)		Value
Ultrasound complementation	0.94	(0.66-1.32)	0.72
Age	1.00	(0.98-1.02)	0.78
Female sex	1.02	(0.71-1.47)	0.87
Absence of other joint pain	0.88	(0.57-1.36)	0.59
Initial ESR ≥40 mm	0.83	(0.38-1.82)	0.65

ESR, erythrocyte sedimentation rate; MTX, methotrexate; CRP, C-reactive protein

### Propensity-score matched analysis

**Figure 3:**
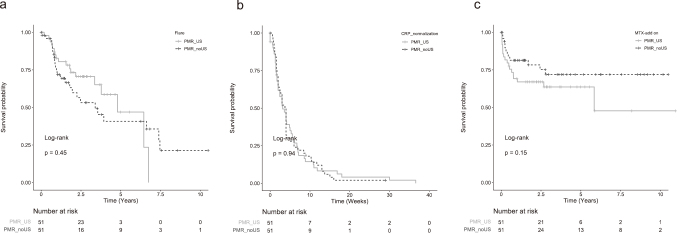
Kaplan-Meier analysis of the patients with and without ultrasound complementation at diagnosis after 1:1 propensityscore matching a. Cumulative incidence of flare. b. Cumulative incidence of CRP normalization. c. Cumulative incidence of MTX add-on MTX, methotrexate; CRP, C-reactive protein; PMR_US, PMR patients diagnosed with ultrasound complementation; PMR_noUS, PMR patients diagnosed without ultrasound complementation

A total of 51 pairs of patients were successfully matched, and covariates including age, CRP level, and absence of other joint pain reached equilibrium in both groups (p>0.05) (Table 1). After determining bias scores, PMR_US and PMR_noUS groups showed a similar risk of flare (p=0.45), MTX add-on (p=0.15), and CRP normalization (p=0.94) (Figure 3).

## Discussion

We retrospectively examined the impact of ultrasound complementation at PMR diagnosis and its impact on the clinical course after GC introduction by comparing PMR_US and PMR_noUS groups. Ultrasound complementation was utilized for patients with certain characteristics and showed a similar impact on the clinical course compared with that observed in patients diagnosed with PMR by only clinical determination.

Ultrasound examination may increase the specificity in diagnosing PMR by discarding mimics, such as infections, malignancy, systemic vasculitis, RA, connective tissue diseases, late-onset undifferentiated spondyloarthritis, hypothyroidism, fibromyalgia, osteoarthritis, gout, pseudo-gout, and musculoskeletal shoulder conditions^16)^. We showed that approximately half the patients with clinically suspected PMR were diagnosed with other diseases, indicating a higher diagnostic accuracy than just clinical diagnosis. Conceivably, the purity of PMR might be associated with a better clinical course after the introduction of GCs. Previous ultrasound studies showed inconsistent results regarding the predictive power of the clinical course. Studies conducted one decade ago showed the usefulness of ultrasound. In one study, patients with evidence of a power Doppler signal in the shoulders at baseline had a higher frequency of relapse than that observed in patients without this evidence^4)^. In addition, the presence of ultrasound findings at baseline was shown to predict the response to glucocorticoids at 4 weeks^11)^. Meanwhile, recent studies showed inapplicability of ultrasound assessments. A previous study found that remission and relapse were not associated with baseline articular ultrasound findings^6)^. Another study suggested that ultrasound findings did not significantly differ between PMR patients with or without recurrences^12)^. Female sex, peripheral arthritis, acute-phase reactant levels, starting GC dosage, and the tapering pace of GCs were the most frequently investigated potential predictors of clinical course; however, lengthy GC treatment and relapse can yield inconsistent results^7)^. To validate ultrasound's usefulness in prognosis rather than diagnosis, we introduced a control group. However, both groups showed similar clinical courses, adding to the evidence that baseline ultrasound complementation may not affect the clinical course.

The shoulders and hands may be inappropriate joint regions for ultrasound screening to determine a better clinical course in PMR. An FDG-PET/CT scanning study revealed that the incidence rates of extra-articular and articular involvement in PMR were increased in the hip (100%), shoulder (97.5%), knee (96.2%), and extra-articular synovial structures (91.4%)^17)^. A whole-body MRI scanning study showed a characteristic pattern of symmetrical, extracapsular inflammation in hip and pelvic regions, and the area adjacent to the greater trochanter as well as the acetabulum, ischial tuberosity, and symphysis pubis were associated with complete GC response (p=0.01), higher pre-treatment CRP and serum IL-6 levels, and better post-treatment function and fatigue outcomes than those in other regions^18)^. These results indicate that global, exhaustive joints and extra-articular regions should be examined, with a particular focus on the hip and pelvic regions, to predict the PMR prognosis. The bulk of inflammation and PMR's characteristic distribution in the body might not be sufficiently revealed by an ultrasound of only the shoulders and hands, as in this study. A systemic ultrasound body survey is required, although it might not be feasible in real-world clinical settings.

This study identified three clinical factors that indicate the need for ultrasound complementation to establish a diagnosis of PMR: age, CRP level, and involved joint distribution. Among older patients, PMR must be differentiated from elderly-onset RA, which has similar clinical features and large joint involvement and is seronegative for rheumatoid factor and anti-cyclic citrullinated protein antibodies. High CRP could be indicative of an infection that might cause similar physical symptoms and should be ruled out. Elevated CRP reflects general inflammation; thus, ultrasound could be useful to validate the degree of inflammation to determine the required GC dose. Pain in the shoulder and hip joints is a representative feature of PMR, yet the same characteristic joint pain distribution could also be observed in elderly-onset RA; the differential diagnosis would be difficult to determine based only on clinical examination. Ultrasound complementation may be utilized in these symptomatic patients to reduce the difficulty in making a differential diagnosis.

Our study had some limitations. First, the study design was retrospective; further prospective studies are essential. Second, our study was conducted at a single rheumatology center, and the number of patients was limited. Third, because the ultrasound examination was conducted only on the shoulders and hands to evaluate feasibility in routine clinical settings, systemic multiple-joint evaluation is required for a more accurate validation in the future. Finally, the results of propensity-score matching are generalizable only to patients within the range of propensity scores. Propensity-score methods are subject to biases due to unobserved differences.

In summary, we implemented a retrospective analysis to investigate the impact of ultrasound complementation at PMR diagnosis on the clinical course, compared with PMR diagnosed without ultrasound. Ultrasound complementation was utilized for differential diagnosis in patients with advanced age, elevated CRP levels, and shoulder and hip joint distribution and showed a similar clinical course, flare, MTX add-on, and CRP normalization rates, compared with those in only clinically diagnosed PMR patients. Although ultrasound complementation may facilitate the differential diagnosis, for predicting the clinical course of PMR after GC introduction, systemic ultrasound utilization ─ or another imaging modality, such as FDG-PET/CT or whole-body MRI scanning ─ may be more informative. Further large prospective investigations are needed.

## Funding

No funding was received.

## Author contributions

TK collected and analyzed the data and assisted in preparing the manuscript. MO designed the study concept, analyzed the data and prepared the manuscript. All authors other than above critically reviewed the manuscript. MMY, KT, YK and NT supervised the study. All authors read and approved the final manuscript.

## Conflicts of interest statement

The authors declare that there is no conflicts of interest.
